# Oncological monitoring after transanal total mesorectal excision for rectal neoplasia

**DOI:** 10.1007/s10151-023-02866-3

**Published:** 2023-10-09

**Authors:** M. Gachabayov, R. Bergamaschi, H. Wasmuth, A. Faerden, M. Javadov, F. Cianchi, M. Barnajian, D. E. Popa, H. Lee

**Affiliations:** 1 Section of Colorectal Surgery, Westchester Medical Center, New York Medical College, Valhalla, NY USA; 2 Department of Gastrointestinal Surgery, St Olav’s Hospital, Trondheim University Hospital, Trondheim, Norway; 3 Department of Digestive Surgery, Akershus University Hospital, Akershus, Norway; 4 Department of Surgery, Yeditepe University Hospital, Istanbul, Turkey; 5 Department of Surgery and Translational Medicine, Careggi Hospital, University of Florence, Florence, Italy; 6 Division of Colorectal Surgery, Cedar Sinai Medical Center, Los Angeles, CA USA; 7 Department of Surgery, Linköping University Hospital, Linköping, Sweden; 8 Division of Colon and Rectal Surgery, Harbor-UCLA Medical Center, Torrance, CA USA

Dear Sir,

We read with great interest the study by Sanchon et al. of 100 patients who underwent trans-anal total mesorectal excision (taTME) although they were neither obese (median BMI 26.9 kg/m^2^) nor presented with cancer of the distal third of the rectum (median distance from anal verge 7.6 cm) [1]. We would like to contribute to the external validity of the core message by adding the following comments.

We take issue with the authors’ assertion that “One of the advantages of the trans-anal route is the achievement of better oncologic results.” In fact, the meta-analysis referenced by the authors did not show improvement of the histopathology metrics of trans-anal over trans-abdominal TME [2]. Moreover, three national studies claiming oncological advantages for taTME have been found to suffer from inappropriate patient selection, statistical underpowering, or even miscalculations [3].

Regarding local recurrence rates, Sanchon et al. reported two such cases with multifocal pattern but failed to include the patient cohort that underwent trans-abdominal TME at the same institution during the same period. In fact, it is noteworthy that the Norwegian data identified an increased hazard ratio of 6.71 after Cox regression analysis [4]. Without a comparison group, the authors’ conclusion that taTME is “safe and effective” has little meaning.

Lastly, it is worrying  that Sanchon et al. “did not receive any specific training in trans-anal surgery nor did they receive mentoring during the learning curve”.

## Data Availability

Data sharing not applicable to this article as no datasets were generated or analyzed during the current study.
